# Diffuse large B-cell lymphoma presenting as reversible intrapulmonary arteriovenous shunts with hypoxia, fever and progressive jaundice: a case report and literature review

**DOI:** 10.1186/s12890-022-01881-8

**Published:** 2022-03-15

**Authors:** Huan Hou, Cuiyan Guo, Chengli Que, Ligong Nie, Qi Zhang, Hong Zhao, Lin Nong, Wei Ma, Qian Wang, Zeyin Liang, Bingjie Wang, Jing Ma, Guangfa Wang

**Affiliations:** 1 Department of Respiratory and Critical Care Medicine, Peking University First Hospital, 8 Xishiku Street, Xicheng District, Beijing, 100034 China; 2 Department of Infectious Diseases, Center for Liver Disease, Peking University First Hospital, Beijing, China; 3 Department of Pathology, Peking University First Hospital, Beijing, China; 4 Department of Cardiology, Peking University First Hospital, Beijing, China; 5 Department of Hematology, Peking University First Hospital, Beijing, China

**Keywords:** Case report, Intrapulmonary arteriovenous shunts, Diffuse large B-cell lymphoma, Fever of unknown origin

## Abstract

**Background:**

Intrapulmonary arteriovenous shunts is rare seen in a patient without lung involvement.

**Case presentation:**

This is the first report of reversible intrapulmonary arteriovenous shunts secondary to extrapulmonary lymphoma as one initial symptom. The patient presented as fever of unknown origin and dyspnea, and examinations of infection were negative. Diagnosis of DLBCL was finally confirmed through bone marrow and splenic biopsies. Intrapulmonary arteriovenous shunts were diagnosed through 100% oxygen inhalation test and transthoracic contrast echocardiography (TTCE). After the treatment of lymphoma, his respiratory failure was relieved. We rechecked the 100% oxygen inhalation test and TTCE, which both indicated that his intrapulmonary arteriovenous shunts had resolved.

**Conclusions:**

We speculated the prominent inflammation from active DLBCL was the most possible mechanism associated with the reversible intrapulmonary shunt in this patient. These findings will assist us to better understand the mechanism of intrapulmonary shunts.

## Background

Intrapulmonary arteriovenous shunts are abnormal communications between pulmonary arteries and veins with a low incidence of 2–3/100,000 [1]. Diffuse large B-cell lymphoma (DLBCL) is the most frequent type of lymphoma, accounting for 30–40% of all non-Hodgkin lymphomas [2]. There are several case reports about lymphoma combined with pulmonary arteriovenous fistula (PAVF), including pulmonary mucosal-associated lymphoid tissue lymphoma and Hodgkin’s lymphoma [3, 4], but it is not clear whether the two are related. We report a case with large amount of intrapulmonary arteriovenous shunts as one of the initial presentations of DLBCL without lung involvement.

## Case presentation

On 2021 April 21th, a 43-year-old man presenting as fever of unknown origin (FUO) and hypoxia was referred to our emergency.

The patient had experienced fever and dyspnea 1 year ago. He was a former smoker and had diabetes. His arterial blood gases(ABG) showed pH 7.41, PaCO_2_ 36.6 mmHg, PaO_2_ 65.0 mmHg on room air, accompanied by slightly elevated PCT (0.26 ng/ml), CRP (22.5 mg/L) and ferritin (910.2 ng/ml). There were no positive findings after comprehensive screening of the common etiology of FUO (Table 1). Cephalosporin companied with oseltamivir was ineffective. His body temperature was normal after dexamethasone (5 mg) for two days. In terms of hypoxia, the D-Dimer(1902 ng/ml) was elevated and ventilation perfusion lung scanning showed decreased perfusion in the left upper lobe, but computer tomography pulmonary angiography (CTPA) and chest CT were negative. Finally, anticoagulant therapy was given for 6 months in consideration of the possible pulmonary embolism, and hypoxia and dyspnea were resolved.

**Table 1:** Screening of the common etiology of FUO

Causes of FUO	Inspection item
Infectious diseases	Blood culture, blood mNGS, respiratory pathogens, PPD, brucella agglutination test, widals reaction, CMV/EBV-DNA, the antibody of toxoplasma, rubella virus, herpes simplex virus, hantaan virus, the pathogenic screening of CSF and BM
Malignancy disorders	Tumor marker, superficial lymph node ultrasound, chest CT, abdomen and pelvis CT, PET-CT, BM biopsy
Rheumatologic inflammatory disorders	ANA, anti-dsDNA, anti-ENA, ANCA, anti-GBM, myositis antibodies, vascular ultrasound

mNGS, metagenomic next-generation sequencing; PPD, purified protein derivative; CMV, cytomegalovirus; EBV, Epstein-Barr virus; CSF, cerebrospinal fluid; BM, bone marrow; ANA, antinuclear antibody; ANCA, anti-neutrophil cytoplasmic antibody; anti-dsDNA, anti-double stranded deoxyribonucleic acid antibody; anti-ENA, anti-extractable nuclear antigen antibody; anti-GBM, anti-glomerular basement membrane

**Fig. 1:**
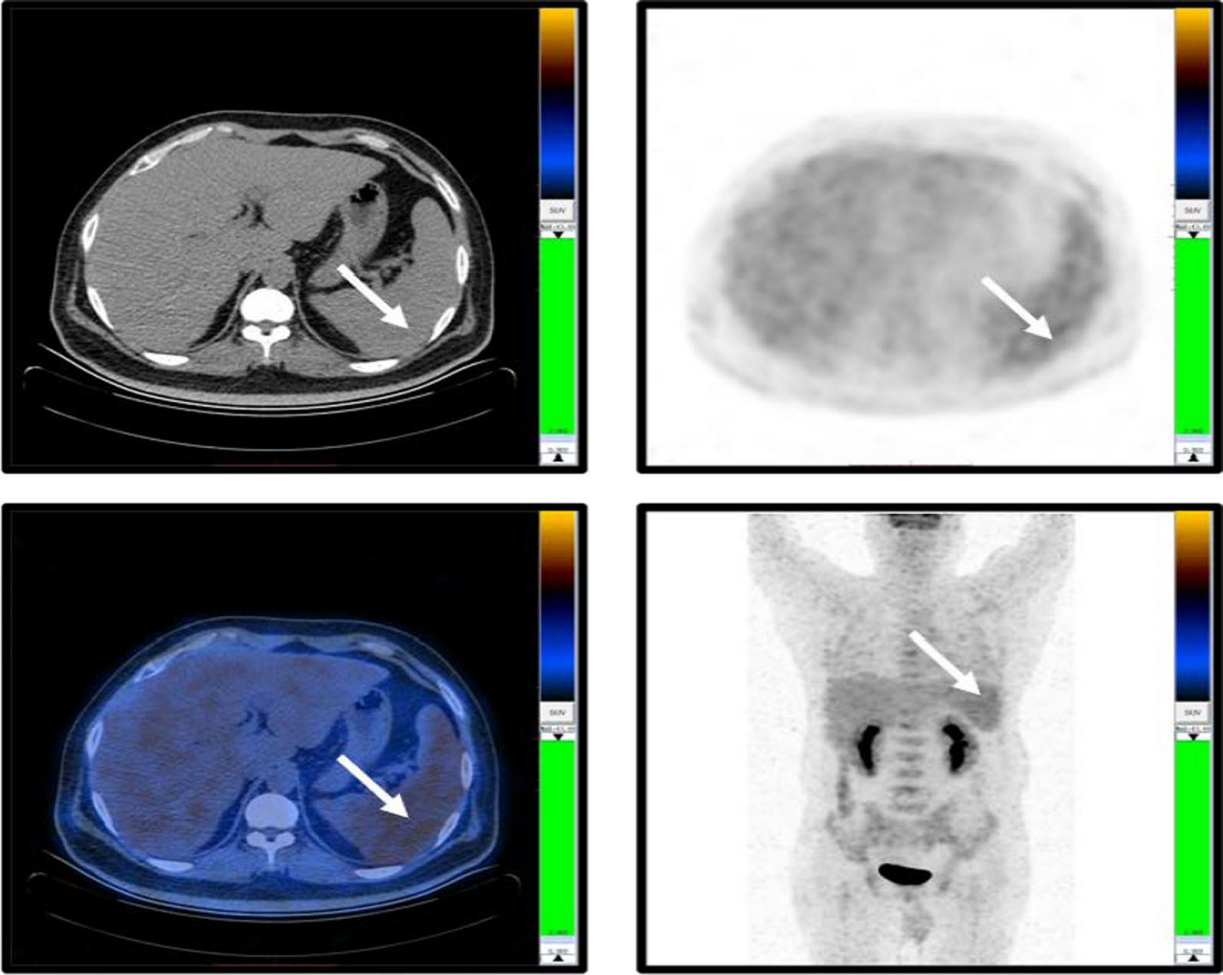
PET-CT images show splenomegaly (about 7 rib units), and slightly increased glucose metabolism (SUV_max_ 4.1)

**Fig. 2:**
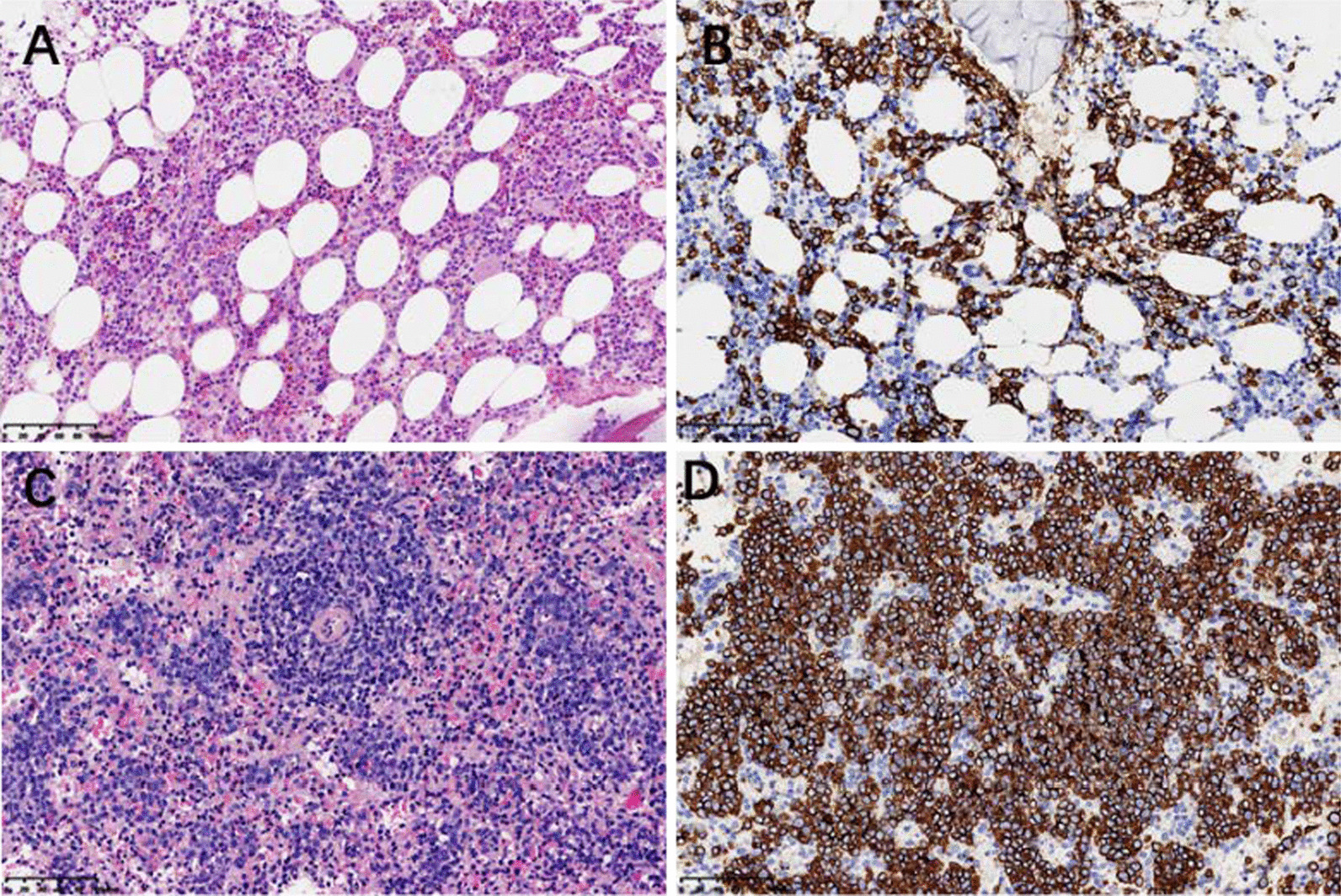
**A** In the interstitium of BM tissue, there were many medium-large heteromorphic lymphocytes scattered or focal infiltration, with round and irregular nuclei. **B** Immunohistochemical staining confirmed that the atypical lymphoid cells in BM were positive for CD20. **C** Large heteromorphic lymphocytes in spleen were infiltrated with foci, uniform cell morphology, ovoid nucleus and obvious nucleoli. **D** Immunohistochemical staining confirmed that the atypical lymphoid cells in spleen were positive for CD20

This time, the patient complained fever and dyspnea again. His ABG showed pH 7.46, PaCO_2_ 32.0 mmHg, PaO_2_ 44.0 mmHg on room air, and the complete blood count, liver enzyme, and bilirubin level was normal in the first inspection in the local hospital. These symptoms did not improve after antibiotic treatment for two weeks. After the patient’s referral to our hospital, we focused on differential diagnosis of FUO. The patient developed slight anemia with hemoglobin of 112 g/L and progressive elevation of CRP (313.2 mg/L), ESR (111 mm/1 h), PCT (3.4 ng/ml), IL-6(404.4 pg/ml), ferritin (1356.1 ng/ml), LDH (557 IU/L), and direct bilirubin level (24.1umol/L). Comprehensive microbiological and autoantibody tests were all negative. Complete course of broad-spectrum antibiotic treatment was in vain. Therefore, we performed abdomen and pelvis CT scan, PET-CT, bone marrow (BM) biopsy, lumbar puncture and further examination (Table 1) to detect potential inflammatory lesions or tumor. Only splenomegaly (about 7 rib units) was found in CT scan with slightly increased glucose metabolism (SUVmax 4.1 in PET-CT) (Fig. 1). BM biopsy suggested aggressive large B-cell lymphoma (Fig. 2A, B), though his initial blood routine examination was approximately normal. In the process, the patient's general condition and blood tests deteriorated rapidly with the total bilirubin and direct bilirubin level increasing to 103.1 μmol/L and 72.8 μmol/L within a week, respectively. But his alanine aminotransferase and aspartate transaminase only slightly elevated by 57 IU/L and 106 IU/L. We performed abdominal ultrasonography and found splenomegaly (16.0 * 6.1 cm) along with hepatomegaly (oblique diameter of right lobe of liver was 17.8 cm). Therefore, we conducted spleen biopsy according to the PET-CT and abdominal ultrasonography, verifying that the spleen was infiltrated by lymphoma either (Fig. 2C, D).

**Fig. 3:**
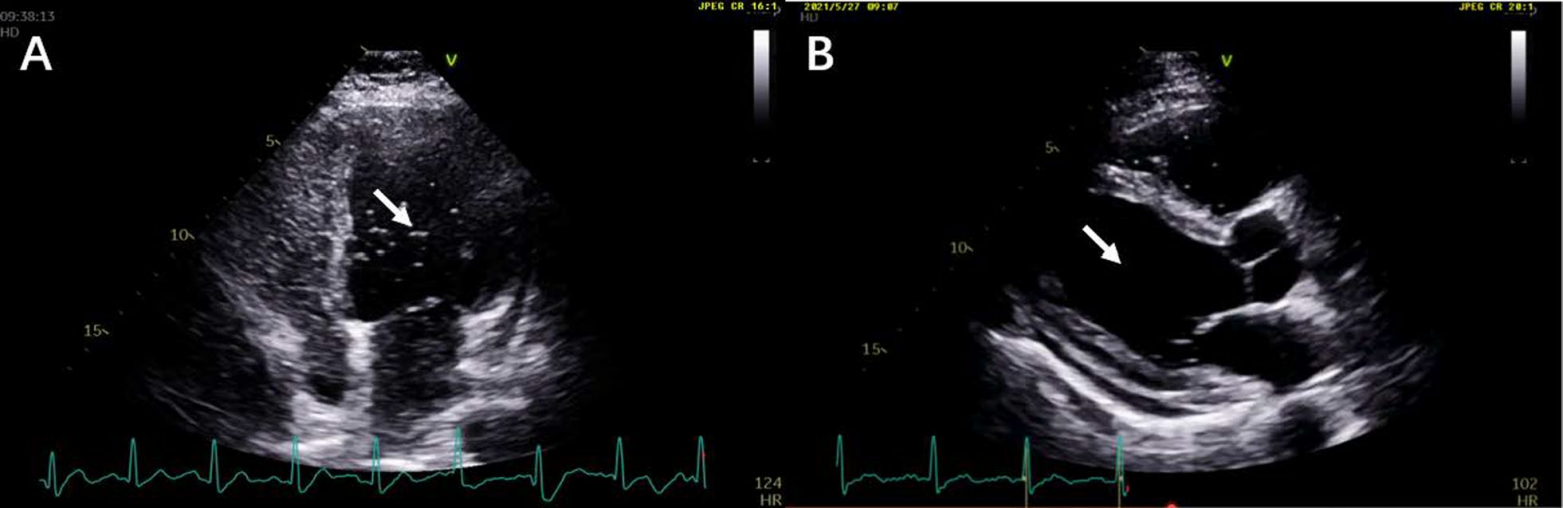
**A** TTCE detected a great deal of right-to-left shunting after 7 cardiac cycles of the right heart images (arrow). **B** rechecked TTCE shown no detection of shunting after 2 courses of chemotherapy for DLBCL

In terms of hypoxic respiratory failure, the D-dimer, CTPA and ventilation perfusion lung scanning were normal. The echocardiography found no obvious cardiac disease, but high cardiac output (CO) (CO: 10.56 L/min, heart rate: 110 bpm, estimated pulmonary artery pressure: 32.6 mmHg). Pulmonary function test showed normal ventilation function and a decreased DLCO (47.4%Pred). Because of no evidence of visible cardiac disease, pulmonary parenchymal and vascular disease, we further performed the 100% oxygen inhalation test and transthoracic contrast echocardiography (TTCE) to determine the possible shunts. The shunt fraction was about 47% (normally less than 5%) and TTCE detected a great deal of right-to-left shunting after 7 cardiac cycles of the right heart imaging (Fig. 3A), indicating the existence of intrapulmonary arteriovenous shunts. Valsalva maneuver in TTCE also did not find a patent foramen ovale (PFO).

After two cycles of R-CHOP (rituximab, cyclophosphamide, doxorubicin, vincristine, and dexamethasone) chemotherapy for DLBCL, the patient’s fever, hypoxia and liver function gradually resolved. ABG showed pH 7.42, PaCO_2_ 40.9 mmHg, PaO_2_ 76.3 mmHg on room air. The level of PCT, CRP and direct bilirubin returned to normal. To investigate the causal link between intrapulmonary shunt and lymphoma in this patient, we repeated the 100% oxygen inhalation test and TTCE. Dramatically, we found the shunt fraction decreased to 3% and no detection of any sign of shunt by TTCE (Fig. 3B), revealing that the patient’s intrapulmonary shunts was caused by his lymphoma.

## Discussion

This is a case of lymphoma with fever, dyspnea and hypoxemia as the initial manifestations. Lymphoma was the most common malignant tumor among all the causes of FUO, accounting for 8.71% [5]. Therefore, despite of mild anemia and normal metabolism of BM from the PET-CT, we conducted the BM biopsy and confirmed the DLBCL in time. In 93% non-Hodgkin's lymphoma, abnormalities in at least one of the blood counts were quite common [6]. FDG-avidity was high in 97% DLBCL patient [7]. A meta-analysis showed that PET-CT had moderate sensitivity (88.7 percent) and high specificity (99.8 percent) for the detection of BM involvement of DLBCL. Approximately 3% patients with BM involvement had a negative PET scan [8]. Thus, for patients with FUO, normal or slight changes of complete blood count or negative PET-CT scan cannot rule out lymphoma, and timely BM biopsy is necessary.

Moreover, DLBCL typically present with a rapidly enlarging symptomatic mass, usually nodal enlargement in the neck or abdomen. Up to a third of DLBCL may arise from extra-nodal sites, most commonly gastrointestinal tract, skin and soft tissues, bone, or genitourinary tract, rarely spleen, liver, lung and others [9]. Primary splenic lymphoma is rare with a reported incidence of less than 1% while primary hepatic lymphoma is 0.4% of all extra-nodal NHL [10, 11]. Jaundice can be the only clinical manifestation of primary hepatosplenic DLBCL in a case report [12]. As progressive jaundice and developed splenomegaly in this patient, we finally conducted spleen biopsy and verified the involvement of the spleen. And the liver infiltration couldn’t be excluded due to clinical manifestation and response to treatment.

The unique manifestation of this case is intrapulmonary arteriovenous shunts as one of the initial presentations of active lymphoma and resolved after two cycles of effective chemotherapy, which proved it was secondary to lymphoma.

As to etiologies of intrapulmonary arteriovenous shunts, most are genetic or congenital, such as hereditary hemorrhagic telangiectasia [13] and large diameter pulmonary arteriovenous malformations. It can also be caused by a variety of acquired disease, with the pathogenesis has not been fully elucidated. It was reported that some conditions can lead to the formation of PAVFs, such as blood metastatic in the lung of choriocarcinoma and renal cell carcinoma [14, 15]. Less common concomitant diseases include penetrating chest trauma, mitral stenosis, schistosomiasis, actinomycosis, Fanconi syndrome, and metastatic thyroid cancer [16]. Previous literature reported pulmonary mucosal-associated lymphoid tissue lymphoma in combination with PAVF but not clarified the relationship. As for our patient, the chest CT and PET-CT didn’t show any visible abnormality in his lung and large vessels, which indicated his shunts might be induced by certain factors.

Intrapulmonary shunt can be inducible and may be reversed by some specific conditions, which is another more important etiology. It is established that intrapulmonary arteriovenous anastomoses exist in the lung and may contribute to gas exchange inefficiency under both physiological and pathophysiological conditions [17]. Previous studies demonstrated these inducible intrapulmonary arteriovenous anastomoses are closed at rest but can open during hyperdynamic conditions such as exercise in healthy humans, as well as hypoxemia, body positioning and catecholamine [18–21]. More importantly, some bioactive substances and cytokines can regulate the patency of the pulmonary arteriovenous communication leading to acute shunting in all lung, which is distinct from structures arising from genetic or surgical induced shunting. Regardless of multiple regulatory mechanisms, the shunting may be chronic by the functional and histological remodeling to shunting vessels ultimately [22]. The typical condition is hepatopulmonary syndrome (HPS) in end-stage liver disease [23]. The accumulation of vasoactive mediators such as NO may lead to pulmonary vasodilation [24], resulting in intrapulmonary shunt, which can be reversed by liver transplantation.

Based on the above theories, considering the patient’s significantly elevated CRP, PCT, and IL-6, we speculated the presence of inflammatory cytokine storm in his rapidly progressive active lymphoma might induced the reversible intrapulmonary arteriovenous shunts. Similarly, Kotwica et al. [25] reported that intrapulmonary shunt predicted worse outcome in COVID-19 and correlated with markers of activated inflammatory response (i.e. CRP, LDH). IL-6 was found to impair hypoxia pulmonary vasoconstriction in animal models and could be inhibited by human recombinant IL-6 and antibody against IL-6 [26, 27]. Therefore, IL-6 was considered to involve in the intrapulmonary shunt [28]. So, it is reasonable to believe that the prominent inflammation from active DLBCL was the most possible mechanism associated with the reversible intrapulmonary shunt in this patient. Meanwhile, the high cardiac output possibly contributed to the shunt to some extent.

Moreover, possible HPS caused by acute liver involvement and severe progressive jaundice may participate in the patient’s shunts. Although HPS is a frequent complication of end-stage liver disease, it was also present in chronic liver disease and acute hepatitis A [29]. Our patient, without past history of liver diseases, developed respiratory failure when the liver function was normal, and did not get worse as liver function deteriorated. So, it is reasonable to think the liver factor might contribute little to the shunt in this patient.

Of note, according to previous studies from animal model [30] and autopsy from patients with COVID-19, Swenson [31] thought microbubbles detected by nonquantitative TTCE might be of trivial importance for gas exchange. He considered the microvascular dilatations represented by the bubbles in these studies were far less than circulation blood flow and the vessel dilation in hepatopulmonary syndrome and not enough to cause hypoxemia. As for this patient, shunt fraction of 47% quantitatively evaluated by 100% oxygen test, without other evidenced causes, was the most possible factor that sufficient enough to result in severe hypoxemia. Furthermore, this was supported by the improvement of hypoxemia accompanied by the decrease of shunt fraction after chemotherapy. Although the significant right-to-left shunt in this patient might explain the decrease of the DLCO in some extent, other potential factors causing hypoxemia could not be completely excluded in this patient, because the DLCO was low at admission and PaO_2_ did not return to normal as the shunt fraction decreasing to normal.

## Conclusion

To our knowledge, this is the first report of reversible intrapulmonary arteriovenous shunts secondary to extrapulmonary lymphoma as one initial symptom. These findings will assist us to better understand the mechanism of intrapulmonary shunts.

## Data Availability

The datasets used and/or analyzed during the current study are available from the corresponding author on reasonable request.

## Abbreviations

ABG: Arterial blood gases
BM: Bone marrow
CTPA: Computer tomography pulmonary angiography
DLBCL: Diffuse large B-cell lymphoma
FUO: Fever of unknown origin
HPS: Hepatopulmonary syndrome
PAVF: Pulmonary arteriovenous fistula
PFO: Patent foramen ovale
TTCE: Transthoracic contrast echocardiography
